# Distinct systemic metabolic phenotypes associated with blood culture positivity and bacterial etiology in community-acquired pneumonia (CAP)

**DOI:** 10.3389/fcimb.2026.1924752

**Published:** 2026-09-25

**Authors:** Mohammad Mehdi Banoei, Sachin Yende, Derek C. Angus, Brent W. Winston

**Affiliations:** 1 Department of Critical Care Medicine, University of Calgary, Calgary, AB, Canada; 2 Department of Biomedical Engineering, University of Calgary, Calgary, AB, Canada; 3 Department of Critical Care Medicine, University of Pittsburgh, Pittsburgh, PA, United States; 4 Center for Research, Investigation, and Systems Modeling of Acute Illness, School of Medicine, University of Pittsburgh, Pittsburgh, PA, United States; 5 Departments of Medicine and Biochemistry and Molecular Biology, University of Calgary, Calgary, AB, Canada

**Keywords:** amino acid metabolism, biomarker discovery, blood culture positivity, community-acquired pneumonia, lipid remodeling, metabolomics, Streptococcus pneumoniae, systemic metabolic response

## Abstract

**Introduction:**

Community-acquired pneumonia (CAP) remains a major cause of morbidity and mortality worldwide, particularly among young children and older adults. Bacterial pathogens account for a substantial proportion of CAP cases, yet early identification of bacteremia and etiologic agents remains challenging. Blood cultures are slow and often have a limited clinical impact. We hypothesized that plasma metabolomics can capture systemic metabolic signatures associated with blood culture positivity and bacterial etiology at the time of hospital admission.

**Methods:**

Plasma samples from 80 patients with bacterial CAP (40 culture-positive and 40 age- and sex-matched culture-negative) were collected on the first day of admission. Metabolomic profiling was performed using complementary platforms, including untargeted ^1^H-NMR, GC-MS, and targeted LC-DIMS/MS. Multivariate and univariate analyses were applied to identify discriminating metabolic features.

**Results:**

Distinct metabolic phenotypes were observed between culture-positive and culture-negative patients across all platforms. OPLS-DA models demonstrated significant discrimination, with GC-MS and 1H-NMR showing strong internally cross-validated discriminatory performance (Q² > 0.45, AUROC > 0.90). Culture-positive CAP was characterized by increased phenylalanine, urea, and mannose, alongside decreased amino acids and fatty acids, indicating coordinated alterations in nitrogen metabolism, energy pathways, and lipid remodeling. Furthermore, metabolomic profiles enabled discrimination of *Streptococcus pneumoniae* from other bacterial causes, with GC-MS and LC-DIMS/MS achieving strong internal discrimination (Q² ≈ 0.4, AUROC > 0.90).

**Conclusions:**

Plasma metabolomics identified distinct systemic metabolic patterns associated with blood culture positivity and, in exploratory analysis, with *S. pneumoniae* etiology. These discovery-stage findings demonstrate reproducible systemic metabolic patterns associated with blood culture positivity and bacterial etiology and support further validation of targeted metabolite signatures in independent CAP cohorts.

## 1 Introduction

CAP is a major cause of morbidity and mortality worldwide and remains a major clinical challenge across all age groups, with the highest fatality rates observed among children younger than 5 years and adults older than 65 years. The worldwide morbidity of CAP is estimated at approximately 0.3–0.5% among adults (Torres et al., 2012). Mortality is influenced by host characteristics, disease severity, and the timing and appropriateness of antimicrobial therapy; therefore, rapid diagnosis and early severity assessment are central to clinical management (Ramirez and Anzueto, 2011; Torres et al., 2012; Remington and Sligl, 2014).

Bacterial pathogens account for approximately 20–50% of CAP cases, although the causative organism often remains unidentified (Remington and Sligl, 2014). *Streptococcus pneumoniae* is consistently reported as the most common bacterial cause, while other typical and atypical pathogens, including *Haemophilus influenzae*, *Staphylococcus aureus*, *Legionella* spp., *Mycoplasma pneumoniae*, and *Chlamydophila pneumoniae*, also contribute to disease burden and may require specialized laboratory testing for detection (Ramirez and Anzueto, 2011; Remington and Sligl, 2014).

Blood cultures are commonly ordered in hospitalized patients with suspected CAP to identify causative pathogens, guide antimicrobial susceptibility testing, and support antibiotic optimization or de-escalation (Mountain et al., 2006; Benenson et al., 2007; Schlueter et al., 2010; Davis et al., 2017; Zhang et al., 2019). However, their clinical utility remains controversial because cultures are positive in only a minority of CAP cases, rarely change early management, and typically require at least 24 hours, and sometimes several days, to provide actionable results (Mountain et al., 2006; Zhang et al., 2019).

Importantly, blood culture positivity in CAP should not be interpreted as a direct marker of a single biological mechanism (Youssef et al., 2020; Zornitzki et al., 2024). Rather, bacteremia likely represents a multifactorial phenotype arising from the convergence of high bacterial burden, inadequate local pulmonary containment, and systemic host-response dysregulation, which may be both a contributor to and a consequence of invasive infection. In this context, plasma metabolomic differences between culture-positive and culture-negative CAP are best viewed as reflecting an integrated systemic response and severity-associated phenotype rather than a purely pathogen-specific signal (Kuang et al., 2025).

Metabolomics enables high-throughput profiling of small-molecule metabolites that reflect integrated systemic metabolic and inflammatory responses to disease. In infectious diseases, metabolomic profiling has been used to identify disease-associated signatures and provide diagnostic and mechanistic insights (To et al., 2015; Fernandez-Garcia et al., 2018; Rees et al., 2018). Prior studies have shown that metabolomics can distinguish bloodstream infection from non-bacteremia by capturing systemic alterations in amino acid, lipid, and energy metabolism associated with bacterial invasion and host immune activation (Fernandez-Garcia et al., 2018). In addition, metabolomics and volatilomics studies have shown that bacterial species can generate distinct metabolic fingerprints under controlled experimental conditions (Rydzak et al., 2022; Olajide et al., 2024; Chen et al., 2025; Zenner et al., 2025). However, translation of species-level discrimination to human plasma remains challenging, particularly in CAP, where host response, comorbidities, and antibiotic exposure may dominate the circulating metabolome (Fernandez-Garcia et al., 2018; Han et al., 2024). In parallel, metabolomics has been used to differentiate bacterial from viral respiratory infections and to identify infection-specific metabolic signatures in pneumonia (Banoei et al., 2017). Building on these findings, we hypothesized that plasma metabolomic profiling obtained on the first day of hospital admission could identify a systemic metabolic phenotype associated with blood culture positivity in CAP. As an exploratory secondary analysis, we also evaluated whether *S. pneumoniae* cases showed distinct organism-associated metabolic patterns compared with other bacterial causes. The objective of this study was therefore to determine whether complementary NMR- and MS-based metabolomics platforms can characterize systemic metabolic phenotypes linked to blood culture status, host-response severity, and selected organism-associated patterns in CAP.

## 2 Methods and materials

### 2.1 Study subjects

In the original cohort, a total of 150 hospitalized patients with bacterial CAP were enrolled at the University of Calgary and the University of Pittsburgh. Inclusion criteria required a clinical diagnosis of CAP and a plasma procalcitonin (PCT) concentration >0.25 ng/mL measured within the first 24 hours of hospital admission, consistent with bacterial infection. Blood samples were collected on the first day of admission before or shortly after initiation of antimicrobial therapy.

A subset of 80 patients was initially identified to be included in the present analysis, including 40 patients with positive blood cultures and 40 age- and sex-matched patients with negative blood cultures. Positive blood cultures were defined as isolation of a pathogenic bacterial species from blood samples by the clinical microbiology laboratory. Negative blood cultures were defined as the absence of detectable organism growth following standard clinical culture procedures.

### 2.2 Analytical platforms

Plasma metabolomic profiling was performed using a combination of analytical platforms that have been described previously in this CAP cohort (Banoei et al., 2020). Three complementary metabolomics platforms were applied: untargeted proton nuclear magnetic resonance (^1^H-NMR) spectroscopy, untargeted gas chromatography–mass spectrometry (GC-MS), and targeted direct infusion liquid chromatography tandem mass spectrometry (LC-DIMS/MS). The integration of these platforms was intended to provide broad metabolic coverage, including low-molecular-weight metabolites, organic acids, amino acids, fatty acids, acylcarnitines, phospholipids, and sphingolipids.

### 2.3 Metabolomics profiling

Three analytical platforms (^1^H-NMR, GC-MS, and LC-DIMS/MS) were applied to plasma samples obtained from patients with bacterial CAP, as previously described in related studies using this cohort (Rees et al., 2018). For ^1^H-NMR analysis: Plasma samples (200 µL) were thawed at 4 °C and filtered using prewashed 3-kDa NanoSep centrifugal filters at 12,000 × g for 1 h at 4 °C. The filtrate was rinsed with 100 µL D_2_O and adjusted to 400 µL using phosphate buffer (0.5 M NaH_2_PO_4_, pH 7.0), DSS as an internal reference, sodium azide, and D_2_O. The final DSS concentration was 0.5 mM and sample pH was adjusted to 7.0 ± 0.04. One-dimensional ¹H-NMR spectra were acquired using a 600-MHz Bruker Ultrashield Plus spectrometer with the Bruker noesypr1d presaturation pulse sequence, water suppression, a 100-ms mixing time, and 1024 scans. Spectra were zero-filled and Fourier transformed to 128k points and processed in TopSpin with phase, baseline, line-broadening, and DSS-referencing corrections. Selected samples were additionally analyzed by TOCSY and ¹H-¹³C HSQC to verify metabolite assignments (Weljie et al., 2006). ^1^H-NMR spectra were profiled using ChenomX NMR Suite version 7.1 (ChenomX Inc., Edmonton, Alberta, Canada) for metabolite identification and quantification (Weljie et al., 2006).

Untargeted GC-MS analysis was performed using an Agilent 7890A gas chromatograph (Agilent Technologies, USA) coupled to a Waters GCT time-of-flight mass spectrometer (Waters Corp., USA), as previously described. For metabolite extraction, 50 µL of plasma was processed using a chloroform-methanol-water extraction procedure. The aqueous phase was dried and derivatized by methoxyamination using methoxyamine hydrochloride in pyridine, followed by silylation with N-methyl-N-(trimethylsilyl)trifluoroacetamide (MSTFA). Derivatized samples were diluted with hexane and centrifuged before GC-MS analysis. Mass spectra were acquired over an *m/z* range of 50–800 (Banoei et al., 2017). Raw chromatograms were processed using MetaboliteDetector software (version 2.06) for peak detection, deconvolution, alignment, integration, and compound identification. Metabolites were annotated by comparison with the Golm Metabolome Database and NIST 2008 mass spectral library (Hiller et al., 2009). The GC-MS workflow detected 185 metabolic features, including 85 structurally annotated metabolites and 100 unannotated features.

Targeted quantitative metabolomics was performed using liquid chromatography direct infusion tandem mass spectrometry (LC-DI-MS/MS) on an ABI 4000 QTRAP mass spectrometer (Applied Biosystems/MDS Analytical Technologies, Foster City, CA) equipped with an integrated solvent delivery system. Targeted analysis was conducted using the AbsoluteIDQ™ p150 kit (BIOCRATES Life Sciences AG, Innsbruck, Austria), which enables the quantification of 151 metabolites, including acylcarnitines, glycerophospholipids, sphingolipids, amino acids, and biogenic amines, according to the manufacturer’s protocol.

Overall analytical coverage differed across the three complementary platforms. The ¹H-NMR workflow identified and quantified 55 low-molecular-weight metabolites, including amino acids, organic acids, sugars, and related metabolites. GC-MS detected 185 metabolic features, comprising 85 structurally annotated metabolites and 100 unannotated features. The targeted LC-DI-MS/MS platform quantified 151 metabolites, including amino acids, biogenic amines, acylcarnitines, glycerophospholipids, sphingomyelins, and hexose. Together, these platforms provided complementary coverage of the circulating metabolome, with partial overlap in the metabolites measured.

### 2.4 Data analysis

Multivariate and univariate statistical analysis were applied to evaluate metabolic differences between CAP patients with positive and negative blood cultures. Prior to multivariate modeling, data were log-transformed and mean-centered, and Pareto scaling was applied where appropriate to reduce the influence of large variance metabolites, consistent with our previous metabolomics studies (Sun and Xia, 2024). For the ¹H-NMR dataset, multivariate analyses were performed using metabolite concentrations identified and quantified by ChenomX profiling, rather than binned spectral intensities.

Principal component analysis (PCA) was first performed as an unsupervised method to obtain an overview of the data structure and to assess intrinsic variability, clustering trends, cohort aggregation, and potential outliers across samples. PCA was used solely for exploratory analysis and quality assessment and not for group discrimination.

Supervised orthogonal partial least squares–discriminant analysis (OPLS-DA) was subsequently applied to model and discriminate between CAP patients with positive and negative blood cultures based on metabolomic profiles. Model performance was evaluated using the goodness-of-fit parameter (R²Y) and the goodness-of-prediction parameter (Q²Y), obtained through cross-validation (CV). Internal validation was performed using leave-one-out cross-validation (LOOCV), as described in our previous work (Cheng et al., 2021). A Q² value ≥0.3 was considered indicative of acceptable predictive performance for models derived from human clinical samples.

To assess model robustness and to exclude overfitting, permutation testing was conducted using 200 random permutations, with statistical significance defined by the distribution of permuted Q² and R² values relative to the original model. Variable importance in projection (VIP) scores were calculated from OPLS-DA models to identify metabolites that contributed most significantly to group separation in a weighted manner. Metabolites with VIP scores >1.0 were considered influential contributors to discrimination. Coefficient plots were generated to visualize the direction and relative magnitude of metabolite associations, indicating metabolites that were increased or decreased in culture-positive compared with culture-negative CAP patients.

Model performance was assessed by calculating sensitivity, specificity, and the area under the receiver operating characteristic curve (AUROC). All multivariate analyses were conducted using SIMCA-P software (version 15.0.2). Correlation analysis was performed to investigate associations between metabolite levels and clinical variables across the full metabolomics dataset. Given the non-normal distribution of several metabolites and clinical parameters, non-parametric Spearman rank correlation coefficients (ρ) were calculated. This approach was selected to capture monotonic relationships without assuming linearity or normality, consistent with previous metabolomics studies.

Correlation analyses were conducted between individual metabolites and key clinical variables, including demographic factors, laboratory measurements, disease severity scores (e.g., APACHE III and Pneumonia Severity Index), and clinical outcomes. Correlations were calculated using normalized metabolite concentrations derived from all analytical platforms. To account for multiple testing, p-values were adjusted using false discovery rate (FDR) correction based on the Benjamini–Hochberg procedure, with adjusted p-values < 0.05 considered statistically significant.

## 3 Results

### 3.1 Patient and cohort characteristics

Eighty patients with community-acquired pneumonia were included, comprising equal numbers of culture-positive and age- and sex-matched culture-negative cases (**Table 1**). Baseline demographic characteristics, including age, sex, body weight, smoking status, alcohol use, and pregnancy, were comparable between groups.

**Table 1:** Baseline demographic and clinical characteristics of the study population stratified by culture status.

Patients’ characteristics	Culture positive (n=40)	Culture negative (n=40)	*P-*value
Age	77.7 ± 8.8	79.2 ± 6.8	0.515
Sex (male/female)	21/19	22/18	0.459
Hospital LOS	10.6 ± 9.1	7.04 ± 3.6	0.014*
ICU LOS	1.6 ± 3.05	1.19 ± 2.6	0.389
Weight	144.6 ± 46.6	159.4 ± 35.8	0.377
APACHE III day1	75.5 ± 22.4	60.8 ± 14.7	0.078
Time 0_PSI	97.9 ± 57.3	72.5 ± 51.3	0.748
Day 1_PSI	136.4 ± 41.0	113.0 ± 27.5	0.013*
Day 1_PSI No Age	63.8 ± 40.7	38.5 ± 24.8	0.002*
Smoking	20 (50 %)	23 (57.5%)	0.243
Pregnancy	5 (12.5%)	8 (20%)	0.307
Alcohol	10 (25%)	13 (32.5%)	0.337
Comorbidity			
Respiratory disease	20 (50.0%)	15(37.5%)	0.160
Neoplastic disease	4 (10.0%)	2 (5.0%)	0.367
Neurological disease	8 (20.0%)	6 (15.0%)	0.365
AIDS	1 (2.5%)	0 (0%)	0.494
Sepsis	14 (35.0%)	11 (27.5%)	0.310
Liver disease	1 (2.5%)	0 (0%)	0.525
CHF	10 (25.0%)	5 (12.5%)	0.166
Cerebro- Disease	5 (12.5%)	4 (10.0%)	0.565
Renal disease	4 (10.0%)	3 (7.5%)	0.557
Altered Mental Condition	7 (17.5%)	1 (2.5%)	0.041*

Patients were categorized into culture-positive (n = 40) and culture-negative (n = 40) groups. Continuous variables are presented as mean ± standard deviation or median (interquartile range), as appropriate, and categorical variables are expressed as counts (percentages). Comparisons between groups were performed using Student’s *t*-test or Mann–Whitney *U* test for continuous variables and χ² test or Fisher’s exact test for categorical variables, as appropriate. A two-sided *p*-value < 0.05 was considered statistically significant.

Markers of clinical severity showed selective differences. Clinical severity was assessed using the Acute Physiology and Chronic Health Evaluation III (APACHE III) and Pneumonia Severity Index (PSI) scores, with higher scores indicating greater illness severity. Although APACHE III scores at admission tended to be higher in culture-positive patients, this difference did not reach statistical significance. Initial PSI scores at presentation were similar between groups; however, culture-positive patients demonstrated significantly higher PSI scores on day 1, including when age was excluded from the PSI calculation, indicating greater early clinical deterioration.

Hospital length of stay was significantly longer in culture-positive patients, whereas ICU length of stay did not differ between groups. The prevalence of most comorbid conditions was comparable across cohorts, except for altered mental condition, which was more frequent among culture-positive patients.

### 3.2 Differentiation of culture-positive from culture-negative

PCA was first applied as an unsupervised overview of global metabolomic variability across culture-positive and culture-negative CAP patients. As expected in a heterogeneous clinical cohort, PCA primarily captured inter-individual variation, with partial overlap between groups; however, modest grouping trends were observed and were more apparent in the three-dimensional PCA views (**Supplementary Figure S1**). PERMANOVA supported a statistically significant, though small, cohort effect in the ^1^H-NMR and GC-MS datasets, with a stronger group contribution in the DI-LCMS/MS dataset, indicating that blood culture status contributes reproducible multivariate structure across platforms despite not being the dominant source of variance (**Supplementary Figure S1**).

To more directly characterize metabolic differences associated with blood culture status, supervised orthogonal partial least squares–discriminant analysis (OPLS-DA) models were constructed independently for the ^1^H-NMR, GC-MS, and LC-DIMS/MS datasets (**Figure 1**). The ¹H-NMR model was based on the quantified ChenomX metabolite concentrations rather than binned spectral intensities.

**Figure 1:**
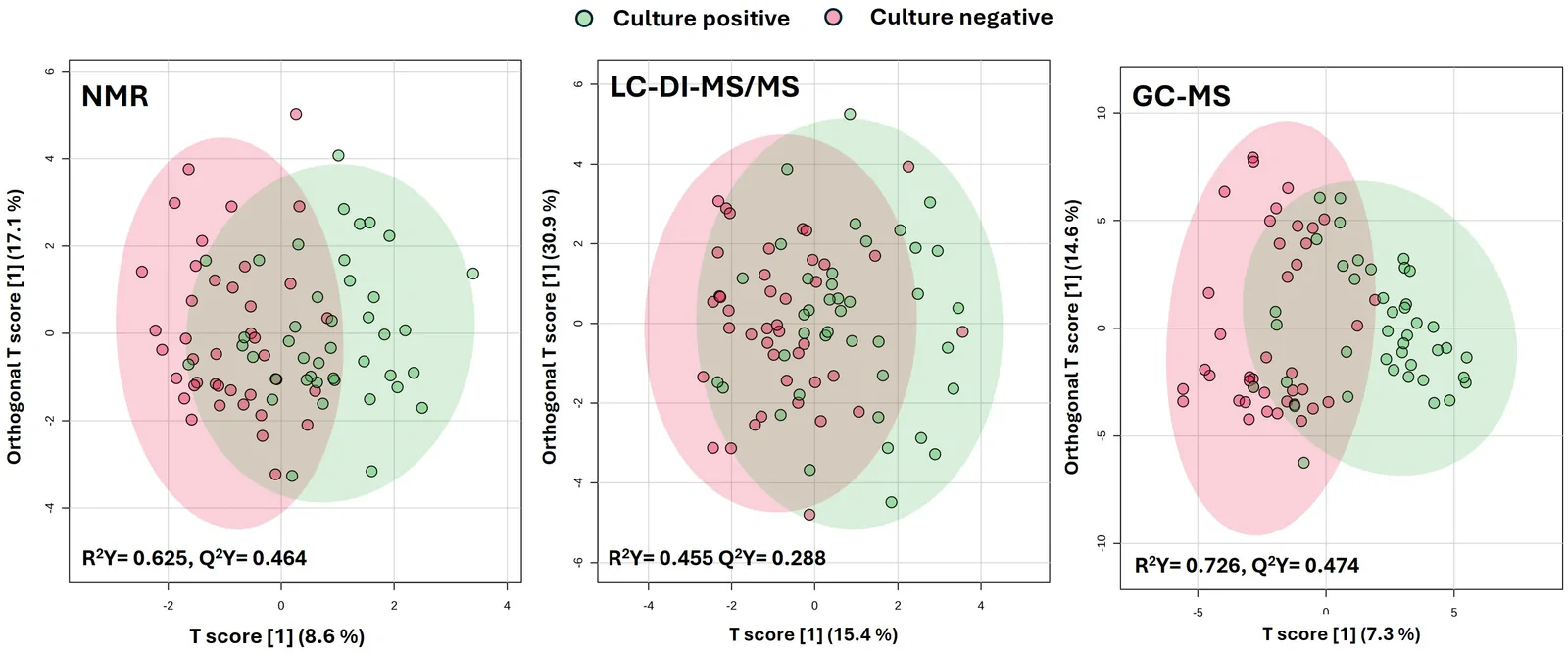
Multivariate score plots (OPLS-DA) illustrating the separation between patients with culture-positive and culture-negative. Score plots were generated using supervised multivariate analysis, with each point representing an individual patient sample. Samples are colored according to culture status (green: positive culture; pink: negative culture). The shaded ellipses indicate the 95% confidence regions for each group, emphasising group clustering and overlap. The percentages indicated on each axis correspond to the explained variance of the respective components.

Across all platforms, OPLS-DA score plots showed improved discrimination between culture-positive and culture-negative patients compared with PCA, while retaining some degree of overlap consistent with clinical heterogeneity. Model performance and robustness were validated through cross-validation and permutation testing, with detailed parameters summarized in **Table 2**.

**Table 2:** The table summarizes the performance and composition of the multivariate models.

Differentiation of Positive Blood Culture from Negative Blood Culture							
Analytical method	R²Y	Q²Y	*p value*	Sensitivity	Specificity	AUROC	#Metabolites
NMR	0.625	0.464	4.6×10^−8^	> 99%	75%	0.94	28
GC-MS	0.726	0.475	1.7×10^−7^	> 99%	> 99%	0.99	71
DI-LC-MS/MS	0.455	0.288	0.0004	85%	77%	0.86	29
Differentiation of *S. pneumoniae* from Other Bacterial Causes of CAP							
Analytical method	R²Y	Q²Y	*p value*	Sensitivity	Specificity	AUROC	#Metabolites
NMR	0.436	0.284	0.004	70%	90%	0.81	20
GC-MS	0.774	0.456	0.003	95%	> 99%	0.97	56
DI-LC-MS/MS	0.657	0.374	0.007	86%	> 99%	0.92	19

Model quality was primarily assessed using Q², with values around 0.3 considered acceptable and values approaching or exceeding 0.5 reflecting cross-validated model performance within the present cohort and should not be interpreted as externally validated clinical predictive performance, while R² reflects goodness of fit. The reported number of metabolites indicates those that most significantly contribute to the optimal models, emphasising the features that drive group discrimination across various analytical platforms using the most differentiating metabolites with VIP >1.0. This integrated presentation aligns with previously validated metabolomics modeling strategies from our group.

Among the three platforms, the GC-MS dataset demonstrated the strongest discriminative model, consistent with its broad analytical coverage and the larger number of discriminative variables it captured. Notably, GC-MS discriminants included both confidently annotated metabolites and high-ranking unannotated (“unknown/no match”) features. In contrast, ^1^H-NMR and LC-DIMS/MS primarily reflected identified metabolites within their respective measurement spaces. LC-DIMS/MS and ^1^H-NMR models also demonstrated statistically significant discrimination, with LC-DIMS/MS showing moderate predictive performance and ^1^H-NMR capturing complementary variation driven largely by low-molecular-weight metabolites. VIP analysis identified sets of discriminating metabolites within each platform (**Table 2**), which were used for subsequent fingerprint definition, cross-platform comparison, and correlation analysis with clinical data variables. **Table 2** summarizes the performance of the OPLS-DA models for discriminating culture-positive from culture-negative CAP across platforms. All three models were statistically significant and demonstrated cross-validated discrimination within the study cohort. This sensitivity, specificity, and AUROC estimates represent internal model performance and were not evaluated in an independent validation cohort, using the GC-MS dataset, followed by the NMR dataset. The LC-DIMS/MS model provided more modest discrimination with lower AUROC and reduced sensitivity/specificity relative to the untargeted platforms (**Table 2**).

### 3.3 Metabolite fingerprint differentiating positive and negative blood cultures

To define metabolic features associated with blood culture status, we examined (i) metabolites prioritized by supervised multivariate modeling (VIP > 1.0) and (ii) metabolites/features identified as statistically significant by univariate testing (p < 0.05; FDR-adjusted where applicable) within each analytical platform. Although multivariate and univariate approaches differ in how they rank discriminating variables, both analyses converged on a consistent pattern of metabolic differences between culture-positive and culture-negative CAP patients.

To define the cross-platform metabolic fingerprint associated with blood culture status, we focused on the most discriminating metabolites retained in the OPLS-DA models (VIP >1.0). We identified those that overlapped across two or more analytical platforms with concordant directionality (**Table 3**). Across platforms, the overlap analysis revealed a small set of metabolites that appeared in two or more datasets and, importantly, showed consistent directionality despite differing analytical chemistries (**Table 3**). This cross-platform “fingerprint” was marked by increased levels of urea, mannose, and phenylalanine in culture-positive patients, along with decreased levels of ornithine and several amino acids (including valine, isoleucine, lysine, serine, asparagine, glutamine, and threonine). Additionally, several saturated fatty acids, namely myristic, palmitic, and stearic acids, were lower in culture-positive patients, aligning with the platform-specific discriminant profiles (**Table 3**).

**Table 3:** Cross-platform comparison of discriminatory metabolites identified by metabolomics analyses.

Panel A: culture-positive vs. culture-negative CAP			
Metabolite	¹H-NMR	GC-MS	LC-DIMS/MS
Aromatic amino acids			
Phenylalanine	**↑**	**↑**	**↑**
Nitrogen metabolism			
Urea	**↑**	**↑**	NM
Carbohydrates & energy			
Mannose	**↑**	**↑**	NM
Amino acids			
Ornithine	**↓**	**↓**	NR
Valine	**↓**	**↓**	NR
Isoleucine	**↓**	**↓**	NR
Lysine	**↓**	**↓**	NR
Serine	**↓**	**↓**	**↓**
Asparagine	**↓**	**↓**	NM
Glutamine	**↓**	**↓**	**↓**
Threonine	**↓**	**↓**	**↓**
Fatty acids/lipids			
Myristic acid	NM	**↓**	**↓**
Palmitic acid	NM	**↓**	**↓**
Stearic acid	NM	**↓**	**↓**
Panel B: S. pneumoniae vs. other bacterial causes			
Metabolite	¹H-NMR	GC-MS	LC-DIMS/MS
Aromatic amino acids			
Phenylalanine	**↑**	**↑**	NM
Carbohydrates & energy			
Lactate	**↑**	**↑**	NM
Glucose	**↓**	**↓**	NM
Amino acids			
Serine	**↓**	NR	**↓**
Glutamate	**↓**	**↓**	NM
Fatty acids/lipids			
Oleic acid	NM	**↑**	**↑**
Stearic acid	NM	**↓**	**↓**

Panel A summarizes metabolites differentiating culture-positive versus culture-negative patients, while Panel B summarizes metabolites distinguishing *Streptococcus pneumoniae* infection from other bacterial causes. The table presents the most discriminating metabolites with VIP >1.0 that overlapped across at least two analytical platforms and contributed to the corresponding OPLS-DA discrimination models, with concordant directionality across platforms. Red cells (↑): metabolite increased in the target group. Blue cells (↓): metabolite decreased in the target group. NM = metabolite was not measured by that platform. NR = metabolite was measured by that platform but was not retained among the VIP >1.0 discriminatory variables.

Platform-specific coefficient and VIP plots further supported these patterns (**Supplementary Figures S2-S4**). In the GC-MS dataset, a mix of annotated metabolites and unannotated features contributed to separation, with key annotated discriminants including organic acids and sugars (e.g., pyruvic acid, glyceric acid, lactic acid, fructose, gluconic/glucuronic acids), nitrogen-related metabolites (urea, ornithine), and lipid-related species (e.g., heptadecanoic acid and related hydrocarbon or fatty acid features). Additionally, a substantial number of “no match/no direct match” signals reflected unresolved annotations within the untargeted feature set. In the DI-LCMS/MS dataset, discrimination was primarily driven by lipid classes (multiple phosphatidylcholines, lysophosphatidylcholines, and sphingomyelins), consistent with the targeted coverage of this platform (**Figure 2**).

**Figure 2:**
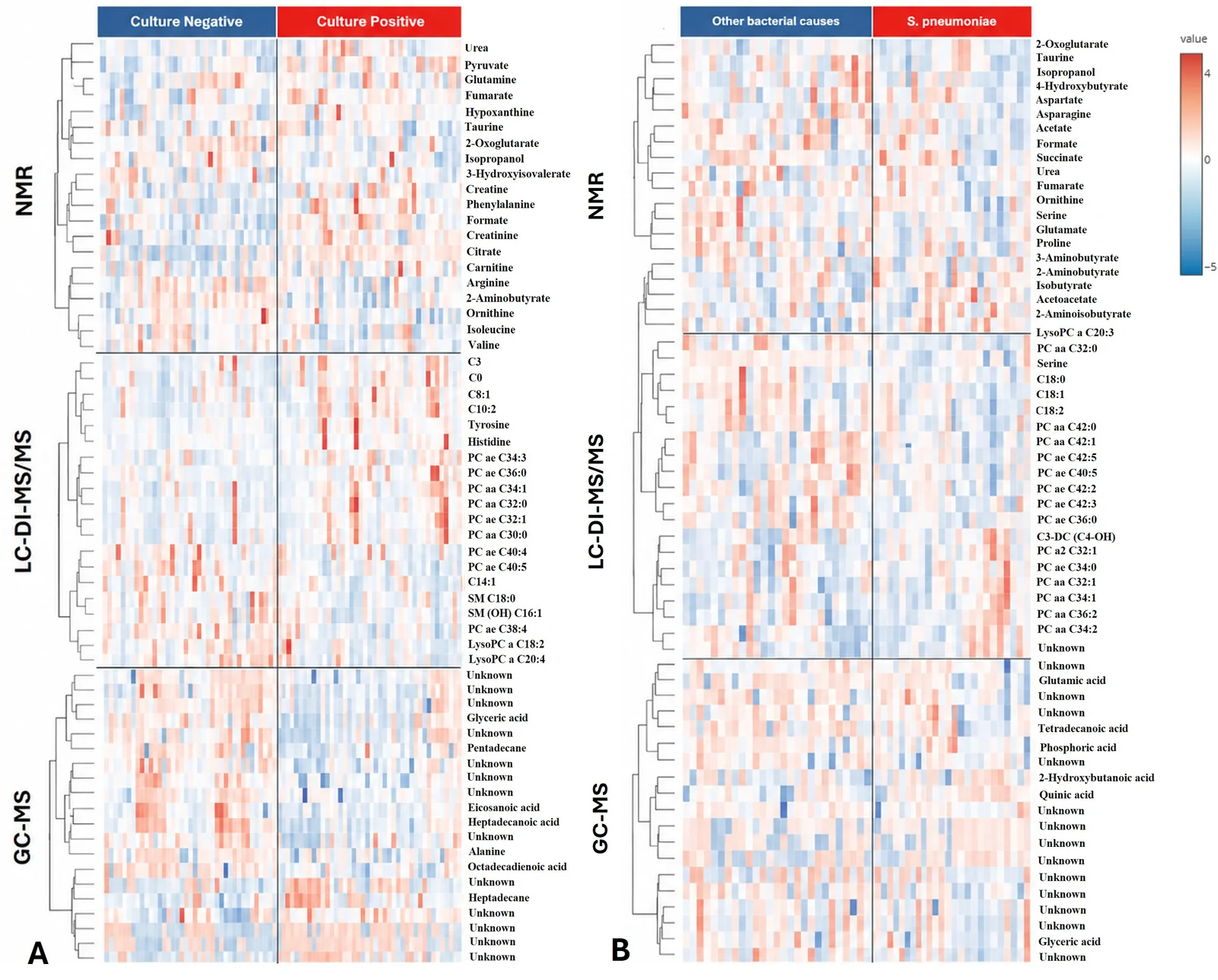
Hierarchical clustering heatmaps illustrating metabolic differences across clinical groups and analytical platforms. **(A)** Heatmap comparing culture-negative and culture-positive patients. Metabolites identified by ¹H-NMR, LC-DI-MS/MS, and GC-MS are shown, highlighting coordinated changes in amino acids, energy-related metabolites, and lipid species between groups. **(B)** Heatmap comparing Streptococcus pneumoniae with other bacterial causes. Distinct clustering patterns across all platforms reveal differences in amino acid metabolism, central carbon intermediates, and lipid composition.

Univariate analysis confirmed these results, identifying 66 significant features that distinguish culture-positive from culture-negative patients (**Supplementary Tables S1-S3**). As expected from differences in analytical coverage, more significant metabolites and features were found in the LC-DIMS/MS and GC-MS datasets compared to the ^1^H-NMR dataset (**Supplementary Tables S1–S3**). These tables list metabolites identified through t-test/Wilcoxon analysis across the ^1^H-NMR, LC-DIMS/MS, and GC-MS datasets. To show the distribution of key discriminating metabolites between groups, box-and-whisker plots were created for each analytical platform (**Supplementary Figures S4–S6**). These plots demonstrate the direction and extent of metabolite differences between culture-positive and culture-negative patients and support the trends seen in the multivariate VIP analysis (**Supplementary Figures S2–S4**). Overall, although the specific variables highlighted by multivariate and univariate analysis were not exactly the same, the overall direction of change for the main discriminating metabolites was very consistent across methods and platforms, underscoring the robustness of the derived metabolic fingerprint (**Figure 1**).

To further evaluate whether the metabolite differences identified by OPLS-DA/VIP and univariate testing were reflected at the global profile level, we generated hierarchical clustering heatmaps for each analytical platform (**Figure 2**). Across datasets, the heatmaps demonstrated coordinated metabolite shifts that were broadly consistent with the previously defined fingerprint, with culture-positive and culture-negative patients exhibiting partially distinct multivariate patterns despite expected heterogeneity within each group.

In the ^1^H-NMR dataset, the heatmap highlighted a consistent pattern involving low-molecular-weight metabolites, including increased urea and phenylalanine in culture-positive patients, accompanied by lower ornithine and reduced branched-chain amino acids (valine and isoleucine), consistent with the univariate boxplot results. In the LC-DIMS/MS dataset, the dominant discriminatory structure was driven by coordinated alterations across lipid classes and acylcarnitines, including clusters of phosphatidylcholines, lysophosphatidylcholines, and sphingomyelins showing structured shifts between culture-positive and culture-negative patients. In the GC-MS dataset, the heatmap revealed a broader, mixed signature that includes annotated organic acids and sugars, lipid-related features, and a significant number of unannotated variables labeled as “no match/no direct match,” aligning with the VIP-ranked feature set (**Figure 2**). Collectively, these heatmap patterns provide an independent multivariate visualization that supports the metabolite alterations observed in the VIP- and t-test–based analyses and further emphasize that culture positivity is associated with coordinated changes across nitrogen/amino acid metabolism, lipid remodeling, and broader GC-MS feature signatures (**Figure 2**).

To provide a direct visualization of selected metabolite-level differences in the main manuscript, representative features from the three analytical platforms are shown in **Figure 3A**. These examples illustrate the direction and magnitude of culture-associated changes across amino acid, energy-related, and lipid metabolites and complement the multivariate and heatmap findings.

**Figure 3:**
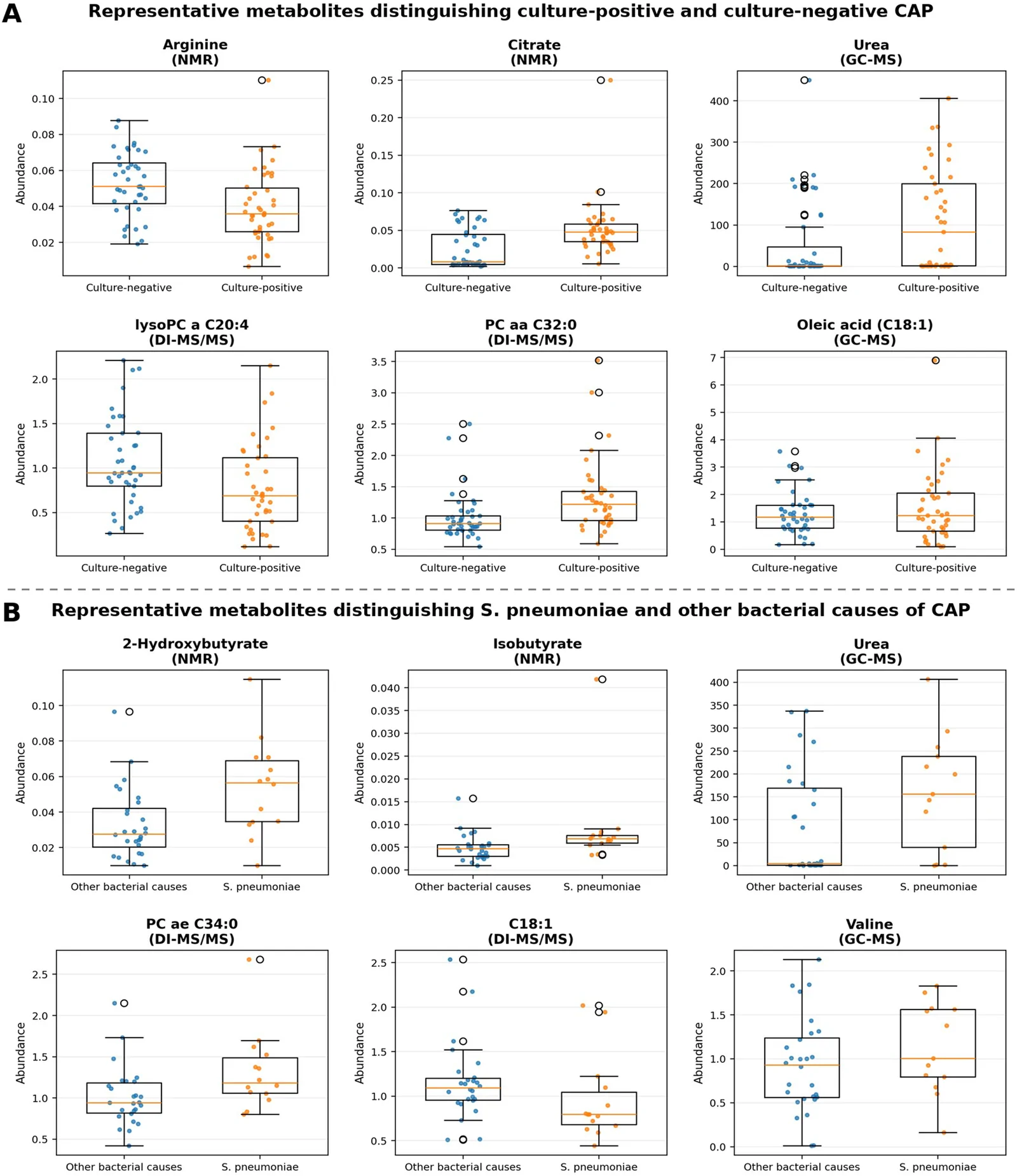
Representative metabolite distributions associated with blood culture status and bacterial etiology in CAP. **(A)** Selected metabolites distinguishing culture-positive and culture-negative CAP. **(B)** Selected metabolites distinguishing *S. pneumoniae*-associated CAP from other bacterial causes. Boxplots show individual patient distributions across ¹H-NMR, GC-MS, and LC-DI-MS/MS platforms.

### 3.4 Exploratory comparison of *S. pneumoniae*-associated CAP with other bacterial causes

We next evaluated whether plasma metabolomic profiles could differentiate CAP cases caused by Streptococcus pneumoniae from those caused by other bacterial pathogens across the three analytical platforms. As expected for a heterogeneous clinical cohort, PCA provided an exploratory overview and suggested modest grouping trends between S. pneumoniae and other bacterial causes, with partial overlap observed across platforms (**Supplementary Figure S7**). These unsupervised patterns inspired supervised modeling to better distinguish metabolic features.

Accordingly, OPLS-DA models were constructed independently for the ^1^H-NMR, GC-MS, and LC-DIMS/MS datasets (**Figure 4**). Across platforms, supervised models demonstrated statistically significant discrimination between *S. pneumoniae* and other bacterial causes, and permutation testing supported the robustness of the models (**Table 2**). Among the three platforms, the GC-MS dataset yielded the strongest overall discriminatory performance, followed by LC-DIMS/MS and ^1^H-NMR, consistent with the broader metabolite coverage captured by GC-MS in this cohort (**Figure 4**, **Table 2**). Importantly, despite differences in analytical coverage and measurement chemistry, the discriminating metabolites identified within each platform showed coherent and reproducible patterns, supporting the presence of a species-associated metabolic signal that can be captured across orthogonal metabolomics technologies (**Figure 2**).

**Figure 4:**
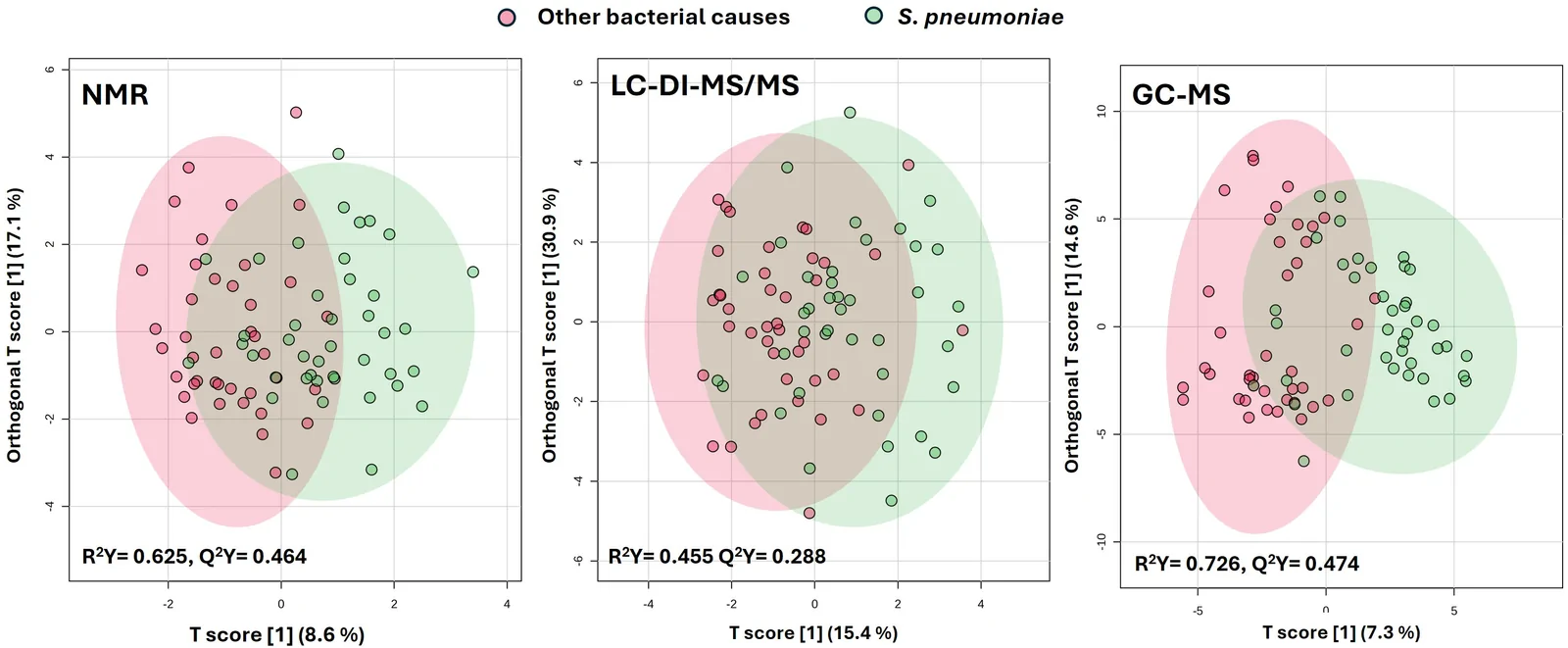
OPLS-DA score plots illustrating the discrimination between *S. pneumoniae* and other bacterial causes across the three metabolomics platforms. ¹H-NMR, GC-MS, and LC-DI-MS/MS. Each point represents an individual patient sample. Samples are colored according to bacterial etiology (green: *S. pneumoniae*; pink: other bacterial causes). The shaded ellipses represent the 95% confidence regions for each group. The percentages shown on the axes represent the explained variance of the respective components.

**Table 2** summarizes the performance of the OPLS-DA models for discriminating *S. pneumoniae* from other bacterial causes across platforms. All three models demonstrated internal discrimination, although these estimates should be interpreted cautiously given the exploratory nature and modest sample size of the etiologic analysis. The GC-MS model showed the strongest overall performance (highest R²Y, Q²Y, and AUROC), with high sensitivity and near-complete specificity, followed by the DI-LCMS/MS model, which also achieved a high AUROC with strong specificity. The ^1^H-NMR model demonstrated more modest discrimination, with lower predictive performance and sensitivity, but maintained high specificity (**Table 2**). Notably, the stronger discrimination observed with GC-MS is consistent with its broader analytical coverage in this cohort, capturing more discriminating features that included both confidently annotated metabolites and high-ranking unannotated (“unknown/no match”) signals (**Figure 2**). In contrast, ^1^H-NMR and DI-MS/MS were largely limited to identified metabolites within their respective measurement panels.

### 3.5 Metabolite patterns associated with *S. pneumoniae* compared with other bacterial causes of CAP

To define a metabolite fingerprint associated with *S. pneumoniae* etiology, we examined the most discriminating variables (VIP > 1.0) derived from OPLS-DA models across the ^1^H-NMR, GC-MS, and DI-MS/MS datasets (**Supplementary Figures S8-S10**). Complementary univariate statistical analysis identified a subset of metabolites that differed significantly between etiologic groups (**Supplementary Tables S4–S6**). These metabolites were visualized using box-and-whisker plots to illustrate their distribution across groups (**Supplementary Figures S11–S13**), confirming the directionality and magnitude of the differences identified in both multivariate and univariate analysis. We evaluated overlap and directional consistency between platforms (**Table 3**). Although each platform captures a distinct biochemical layer, several discriminating metabolites recurred across two or more datasets and showed concordant directionality, supporting a reproducible species-associated metabolic pattern.

Across platforms, *S. pneumoniae* cases were characterized by increased phenylalanine and lactate relative to other bacterial causes, with these metabolites showing consistent directionality where measured (**Table 3**). In contrast, serine and glutamate levels were lower in S. *pneumoniae* compared to other bacteria across the datasets capturing these metabolites. In the ^1^H-NMR and GC-MS datasets, glucose also exhibited a consistent decrease in *S. pneumoniae*, suggesting altered carbohydrate utilization in this subgroup. Lipid-related differences were also observed in the GC-MS and LC-DIMS/MS datasets, with oleic acid increased and stearic acid decreased in *S. pneumoniae* compared with other bacterial causes, indicating species-associated disruptions in fatty-acid and lipid metabolism (**Table 3**).

Platform-specific VIP profiles further supported these findings. The ^1^H-NMR model highlighted a set of low-molecular-weight metabolites, including organic acids and amino-acid–related metabolites, contributing to separation, whereas the LC-DIMS/MS dataset was dominated by phospholipid species (primarily PCs) and related targeted metabolites. The GC-MS dataset captured a mixed signature of annotated metabolites together with high-ranking unannotated features (“no match/no direct match”), consistent with broader feature coverage in untargeted GC-MS. Collectively, these results define a cross-platform metabolite fingerprint for differentiating *S. pneumoniae* CAP from other bacterial causes, centered on coordinated shifts in amino-acid, glycolytic, and lipid-related metabolites (**Figure 2**).

To further evaluate whether the discriminating metabolites identified by OPLS-DA/VIP and univariate testing were reflected at the global profile level, we generated hierarchical clustering heatmaps of the top differentiating metabolites/features for each analytical platform (**Figure 2**). Across datasets, the heatmaps demonstrated coordinated shifts between *S. pneumoniae* and other bacterial causes, with samples showing partial stratification by etiology despite expected overlap within a clinically heterogeneous CAP cohort.

In the ^1^H-NMR dataset, the heatmap revealed a consistent pattern of low-molecular-weight metabolites changes distinguishing *S. pneumoniae* from other bacteria, with several amino acid– and energy-related metabolites showing steady directional shifts across the *S. pneumoniae* samples. In the targeted DI-MS/MS dataset, the dominant discriminatory structure was driven by coordinated alterations across lipid-related metabolites and acylcarnitines, with clusters of PCs/lysoPCs and fatty-acid–related species showing structured differences between etiologic groups. In the GC-MS dataset, the heatmap revealed a broader mixed signature that included annotated metabolites along with a significant number of unannotated (“no match/no direct match”) features that contributed to the discriminatory pattern, aligning with the VIP-ranked feature set from the supervised models.

Importantly, the multivariate heatmap patterns were consistent with the univariate t-test findings, which confirmed significant differences for representative discriminating metabolites across platforms. Collectively, these heatmap visualizations provide independent multivariate evidence that *S. pneumoniae* CAP is linked with coordinated changes in low-molecular-weight metabolites, lipid remodeling, and broader untargeted GC-MS feature signatures, supporting the discrimination seen in the supervised OPLS-DA models. (**Figures 4**, **S11-S13**; **Supplementary Tables S4-S6**).

Representative metabolite distributions for the etiologic comparison are shown in **Figure 3B**, illustrating selected amino acid, organic acid, and lipid features that contribute to the metabolic differences between *S. pneumoniae*-associated CAP and other bacterial causes.

### 3.6 Integrated metabolite–clinical correlation patterns across ^1^H-NMR, LC-DIMS/MS, and GC-MS

To determine whether the metabolic features associated with blood culture status were also related to illness severity, we examined correlations between metabolites and established clinical measures, including APACHE III, day-1 PSI, PSI calculated without age, and hospital and ICU length of stay. Across the three analytical platforms, the strongest metabolite associations were generally observed with measures of disease severity and length of stay, whereas associations with age and sex were less consistent. Because age contributes directly to the standard PSI score, PSI without age was also examined to better distinguish associations with disease severity from those related to age (**Figure 5**).

**Figure 5:**
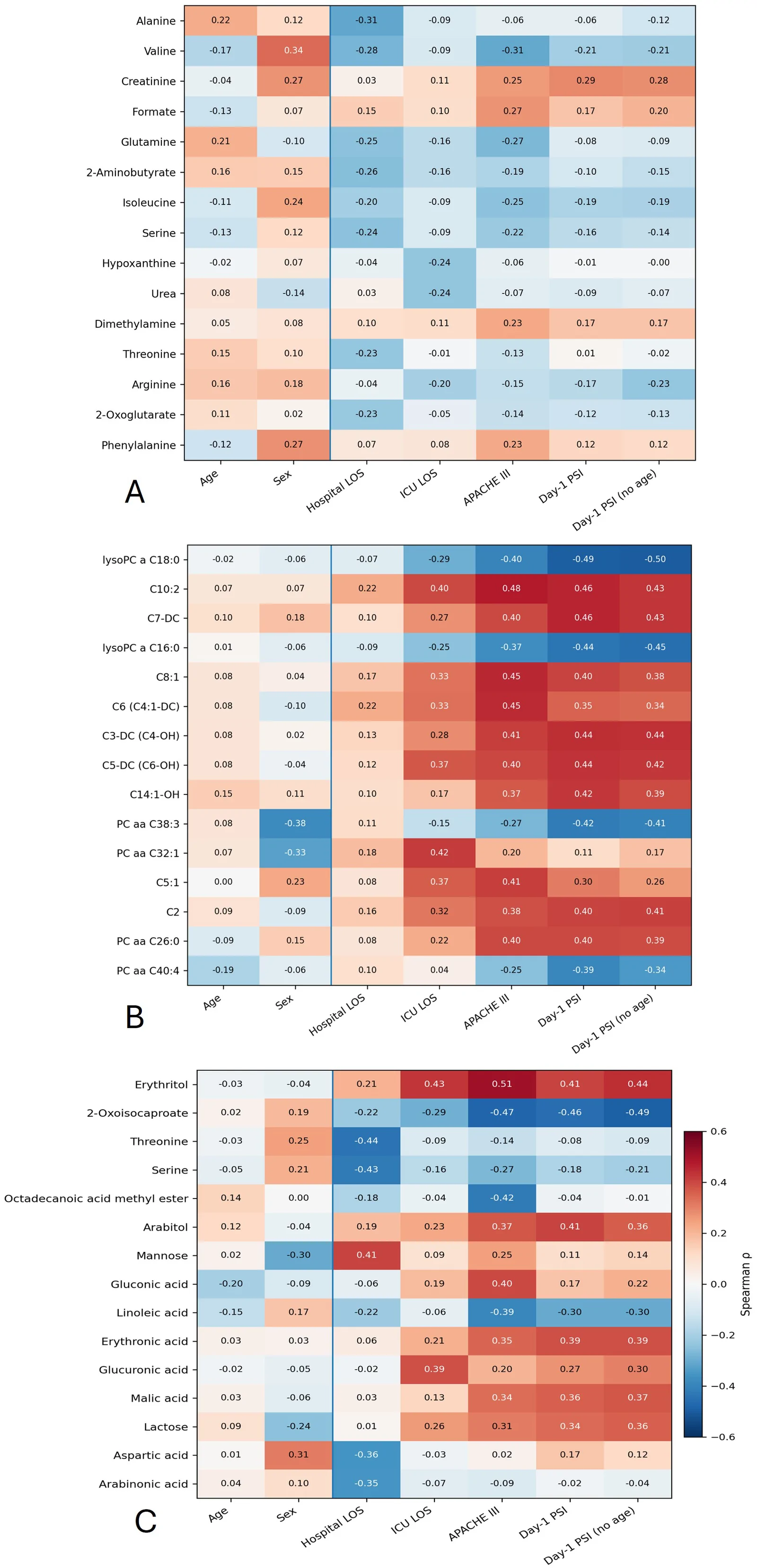
Selected metabolite–clinical correlations across metabolomics platforms. Spearman correlation heatmaps show associations between representative metabolites and clinical severity measures for **(A)** ¹H-NMR, **(B)** LC-DI-MS/MS, and **(C)** GC-MS. Red indicates positive and blue indicates negative correlations, with color intensity reflecting correlation strength (Spearman ρ). Complete metabolite–clinical correlation matrices are provided in **Supplementary Figures S14–S16.**.

In the ¹H-NMR dataset, amino acids and metabolites related to nitrogen and central carbon metabolism showed structured relationships with clinical severity, with branched-chain amino acids including valine and isoleucine generally showing inverse associations with APACHE III and PSI (**Figure 5A**). LC-DI-MS/MS showed coordinated associations involving acylcarnitines, phosphatidylcholines, and lysophosphatidylcholines, with several acylcarnitines positively associated with APACHE III and PSI and selected lipid species showing inverse relationships (**Figure 5B**). GC-MS similarly identified associations involving amino acids, organic acids, sugars/polyols, and fatty-acid-related metabolites (**Figure 5C**).

Overall, the correlation patterns across the three platforms indicate that part of the metabolic phenotype associated with blood culture positivity is also related to disease severity. Several metabolites contributing to culture-status discrimination showed relationships with PSI, APACHE III, or length of stay, supporting a severity-associated component in addition to their association with blood culture status. The complete metabolite–clinical correlation matrices for each analytical platform are provided in **Supplementary Figures S14–S16**.

When the correlation analysis was stratified by blood culture status, metabolite–severity relationships were generally stronger in culture-positive patients, particularly for APACHE III and PSI-derived measures. This pattern was most evident in the LC-DI-MS/MS dataset, where the median absolute correlation across the selected metabolites was higher in culture-positive than culture-negative patients (|ρ| = 0.350 vs. 0.255), with a greater proportion of moderate-to-strong correlations. Similar, although less pronounced, patterns were observed for NMR and GC-MS. For APACHE III, day-1 PSI, and PSI without age, the LC-DI-MS/MS correlations were consistently stronger in culture-positive patients (median |ρ| = 0.415, 0.455, and 0.454, respectively) than in culture-negative patients (0.302, 0.327, and 0.333, respectively). These exploratory findings suggest that the metabolic phenotype associated with blood culture positivity is more closely coupled to the severity of systemic illness. Detailed stratified correlation results are provided in **Supplementary Table S7**.

## 4 Discussion

This study shows that early plasma metabolomic profiling can distinguish community-acquired pneumonia (CAP) patients with positive blood cultures from those with negative cultures and can further differentiate *S. pneumoniae* from other bacterial causes. However, blood culture positivity should be interpreted as a complex clinical phenotype rather than a direct surrogate for a single causal mechanism or pathogen-specific process. Across orthogonal platforms, culture positivity was associated with coordinated disruptions in amino acid metabolism, nitrogen handling, lipid remodeling, and energy/carbohydrate pathways. The detection of these signals at presentation, despite the clinical heterogeneity of hospitalized CAP, supports the concept that plasma metabolomics captures an early systemic phenotype reflecting infection burden and systemic inflammatory and metabolic response intensity before definitive microbiological confirmation (Langley et al., 2013; Seymour et al., 2013; Fernandez-Garcia et al., 2018; Han et al., 2024). Prior pneumonia and sepsis metabolomics studies similarly show that infection syndromes generate reproducible metabolic deviations related to biological response and outcome (Slupsky et al., 2009; Seymour et al., 2013), with lipid-related features repeatedly identified among discriminatory signals in pneumonia phenotypes (Antcliffe et al., 2017). Together, these findings support the biological plausibility that bacterial species and infection intensity can differentially shape the circulating host metabolic state, even when measured in plasma rather than at the site of infection (Seymour et al., 2013; Antcliffe et al., 2017; Han et al., 2024). Mechanistically, the culture-positive metabolic phenotype observed here is consistent with a high-burden inflammatory and catabolic state. Systemic infection increases amino-acid trafficking and protein turnover to support acute-phase protein synthesis, immune activation, and hepatic metabolic demands, while also altering nitrogen handling and energy metabolism as immune and peripheral tissues reprioritize substrate use (Langley et al., 2013; Seymour et al., 2013). Importantly, the diagnostic signal in infection metabolomics often arises from pathway-level coherence across metabolite families rather than extreme changes in single metabolites, which is particularly relevant in heterogeneous clinical cohorts where comorbidities, medications, underlying host gut microbiota and variable sampling times can introduce biological noise (Langley et al., 2013; Seymour et al., 2013; Han et al., 2024).

The lipid remodeling signature is also biologically and clinically relevant. Clinical observations in lower respiratory tract infection indicate that reduced lipid levels, particularly HDL-associated measures, correlate with bacterial infection and adverse outcomes, supporting the idea that systemic infection perturbs lipid transport and lipoprotein biology (Gruber et al., 2009). Lipid classes associated with CAP, including phosphatidylcholines, lysophosphatidylcholines, and sphingomyelins, are integrated into inflammatory signaling, endothelial activation, and immune-cell trafficking networks (Wymann and Schneiter, 2008). Experimental work further links lysophosphatidylcholine (LPC) to inflammatory signaling in vascular cells, while serial LPC measurements have been associated with mortality prediction in severe sepsis and septic shock (Murugesan et al., 2003; Park et al., 2014). Recent plasma lipidomics studies in sepsis due to CAP extend these observations, showing broad remodeling of phosphatidylcholines, lysophosphatidylcholines, and sphingolipids in association with inflammatory burden and disease severity (Chouchane et al., 2024; Kuang et al., 2025; Rischke et al., 2025).

Perturbations in carbohydrate and energy metabolism, including mannose- and glycolysis-related patterns, further support systemic metabolic reprogramming in bacteremic CAP. Acute innate immune activation is closely linked to glycolytic metabolism and coordinated remodeling of amino-acid and lipid pathways, suggesting an integrated response rather than isolated pathway changes (Fernandez-Garcia et al., 2018; Han et al., 2024). In the present cohort, several metabolites and lipid classes that differentiated culture-positive from culture-negative CAP, including phenylalanine, urea, branched-chain amino acids, phosphatidylcholines, lysophosphatidylcholines, and sphingomyelins, also showed structured associations with severity and clinical utilization. This suggests that these features may reflect both infection burden and downstream disease trajectory rather than purely diagnostic effects (Banoei et al., 2020). Longitudinal metabolomic profiling in *S. pneumoniae*-associated CAP further indicates that these metabolic disturbances evolve over the disease course and track clinical recovery or deterioration (Del Borrello et al., 2020; den Hartog et al., 2024). Consistent with this, recent CAP metabolomics studies have reported recurrent involvement of phenylalanine, branched-chain amino acids, acylcarnitines, phosphatidylcholines, and sphingomyelins in models differentiating bacterial CAP from non-bacterial causes or less severe disease (Chen et al., 2024).

Not all metabolic pathways detected in the present study are specific to bacterial infection or bacteremia. Viral respiratory infection involves changes in amino-acid metabolism, central pathways of energy metabolism, and lipid remodeling as well. Our previous study identified some differences between plasma metabolomics of H1N1 influenza pneumonia and culture-positive bacterial CAP, while both conditions exhibited significant metabolic derangements in amino acids, energy-related metabolites, and fatty-acid pathways. The metabolic profiles of patients with ARDS due to COVID-19, H1N1 influenza, or bacterial pneumonia were also shown to be similar and different in more recent targeted metabolomics studies, with alterations in pathways of pyruvate metabolism, glutamate/glutamine metabolism, amino-acid metabolism, and glycerophospholipids. These observations indicate that at least part of the metabolic phenotype described here is perhaps a general response of the body to severe pulmonary infection, while the relative abundance of the individual metabolites may contribute to the variation seen with either bacterial culture status or bacterial etiology. Since the present study was not conducted with a contemporary viral pneumonia comparator, the specificity of the identified metabolic phenotype for bacterial CAP and/or bacteremia remains unknown and should be explored directly in future studies (Banoei et al., 2017; Banoei et al., 2020; Lee et al., 2024).

The pneumococcal-focused analysis further supports biological specificity in CAP. Metabolic profiling of *S. pneumoniae* under infection-relevant conditions has identified conserved reliance on carbohydrate utilization, amino-acid acquisition, nucleotide salvage, and membrane lipid remodeling, all of which are linked to virulence and invasive potential (Passalacqua et al., 2016); Recent targeted metabolomic studies similarly identify glycolytic flux, arginine metabolism, and lipid biosynthesis as central programs supporting pneumococcal adaptation during infection (den Hartog et al., 2024; Jiang et al., 2025). Although these studies examine bacterial metabolism directly, they provide mechanistic context for the plasma signatures observed here, suggesting that alterations in host amino-acid handling, nitrogen metabolism, and lipid pathways may reflect combined effects of bacterial metabolic demand and host immune-driven reprogramming. This interpretation is consistent with multi-omics studies in severe CAP showing coordinated inflammatory activation alongside dysregulated energy, amino-acid, and lipid metabolism (Li et al., 2024).

Notably, a mouse model of pneumococcal pneumonia reported a similar metabolic pattern in lung tissue, including decreased glucose, serine, and glutamate, increased phenylalanine, and alterations in branched-chain amino acid pathways (Green et al., 2023). This concordance between mouse lung and human plasma supports the biological relevance of these metabolic changes in pneumococcal infection.

These mechanistic features have potential clinical relevance because they may inform future diagnostic and risk-stratification strategies. Early identification of bacteremia-likely CAP could support antimicrobial escalation or de-escalation decisions, prompt closer monitoring, and identify patients who may require additional source evaluation. Existing biomarkers such as CRP and procalcitonin are useful but not definitive and are best interpreted alongside clinical severity assessment rather than as standalone tools (Krüger and Welte, 2012; Pereira et al., 2012). Meta-analytic evidence for procalcitonin also emphasizes that its diagnostic value depends on timing and clinical context, reinforcing the need for multi-marker approaches that better capture early systemic biology (Wacker et al., 2013). In this framework, metabolomics-based signatures are not intended to replace clinical judgment or conventional microbiology. Rather, they provide probabilistic molecular evidence that could be integrated with severity scores and established biomarkers, ideally through a compact targeted metabolite panel suitable for more rapid workflows and external validation (Fernandez-Garcia et al., 2018; Han et al., 2024).

Importantly, the present study represents biomarker discovery and should not be interpreted as demonstrating a clinically validated tool. Rather, this study provides a discovery framework for identifying a parsimonious set of metabolites that could be translated into a targeted, clinically deployable assay if and when va,idated. Future development would require reducing the broad metabolomic signature to a small panel of robust metabolites, validating this panel in independent CAP cohorts, and evaluating whether it improves early discrimination of culture-positive CAP beyond conventional clinical variables, CRP, procalcitonin, and severity scores. Such a panel could then be adapted to more rapid analytical workflows, including targeted LC-MS/MS, enzymatic assays, or biosensor-based platforms, with the goal of generating actionable results within a clinically relevant time window. In this way, metabolomics may ultimately complement, rather than replace, blood culture by providing early systemic metabolic information while conventional microbiology remains pending.

It is also important to distinguish the present plasma metabolomics approach from pathogen-centered metabolic diagnostics, such as the metabolic preference assay described by Rydzak et al. That method interrogates pathogen substrate utilization after short ex vivo culture or enrichment to infer bloodstream infection through microbial metabolic activity (Rydzak et al., 2022). This is conceptually complementary but biologically distinct from plasma metabolomics, because circulating metabolite profiles are shaped by host–pathogen interactions, immune activation, organ metabolism, and treatment effects. Thus, while both approaches exploit metabolism as an early signal, ex vivo pathogen metabolic phenotyping focuses on microbial functional traits, whereas plasma metabolomics reflects the integrated systemic response to infection burden and host pathophysiology (Fernandez-Garcia et al., 2018; Rydzak et al., 2022; Han et al., 2024).

Compared with other molecular profiling approaches, metabolomics provides a functional readout that is often closer to phenotype than gene regulation alone. Proteomics studies in CAP and sepsis have identified informative immune and acute-phase proteins and advanced understanding of complement, coagulation, and inflammatory pathways (Langley et al., 2013; Antcliffe et al., 2017). However, many protein biomarkers, including CRP, IL-6, and PCT, may show delayed kinetics or limited specificity for early bacteremia or species-level discrimination (Torres et al., 2012; Faix, 2013; Wacker et al., 2013). Circulating miRNAs have also been proposed as biomarkers in sepsis and pneumonia, but reproducibility can be affected by cell-composition sensitivity, normalization choices, and platform effects, and many studies focus on sepsis versus sterile inflammation rather than blood-culture status or causative species in CAP (Benz et al., 2016; Vasilescu et al., 2017; Valsamaki et al., 2024). These considerations support an integrative strategy in which a targeted metabolite panel could be combined with selected proteins or other molecular markers that add complementary information (Langley et al., 2013; Fernandez-Garcia et al., 2018).

Several limitations should be acknowledged. CAP metabolomics is vulnerable to confounding from comorbidities, medications, antibiotic exposure, host microbiota and variable sampling time relative to symptom onset, all of which may affect generalizability. Antibiotic exposure may reduce microbial-derived signals and shift the circulating metabolome toward host-dominant response biology. In addition, some untargeted features remained unannotated, limiting mechanistic specificity and emphasizing the need for targeted confirmation. Finally, the absence of an independent validation cohort means that the sensitivity, specificity, AUROC, and Q² values reported here represent internal discovery-stage model performance. Although cross-validation and permutation testing reduce the likelihood of simple overfitting, they do not establish generalizability. The discriminatory performance therefore requires confirmation in larger, independent, multicenter CAP cohorts (Fernandez-Garcia et al., 2018; Han et al., 2024). Moreover, the culture-negative comparison group was inherently heterogeneous and may have included patients with localized bacterial infection, partially treated infection, non-detected pathogens, or lower systemic bacterial burden; therefore, organism-level interpretations should be considered exploratory and secondary to the main host-response analysis. Further, a viral pneumonia control group was not assessed, so it is not possible to differentiate between any effects on the observed metabolic phenotype that are specific to bacterial CAP and those that are more general to severe respiratory infection. The plasma metabolomics data do not allow the distinction of the biological origin of the individual metabolites. Metabolites can come either from host metabolism, bacterial metabolism, action of the microbiota, or host–microbial co-metabolism. The relative contribution of these sources could not be determined in this cohort, because microbiome data were not available.

Moreover, sex was well-balanced between culture-positive and culture-negative patients, but the sample was not large enough to describe sex-specific metabolic responses. To study the difference between the metabolic phenotype of blood culture positivity in male and female subjects, larger studies will be needed. In summary, integrated plasma metabolomics identifies a coherent, biologically plausible signature that differentiates culture-positive from culture-negative CAP and separates pneumococcal from non-pneumococcal CAP. The dominant patterns involve coordinated amino acid turnover, nitrogen handling, lipid network remodeling, and energy metabolism, consistent with systemic infection biology and previous metabolomics findings in pneumonia and sepsis. These findings should therefore be considered discovery-stage evidence. Future work should prioritize independent multicenter validation, harmonized pre-analytics, and reduction of the current metabolomic signatures to a compact targeted panel evaluated against CRP, procalcitonin, and established severity scores before clinical implementation is considered.

## Data Availability

The dataset is publicly available at: DOI: https://doi.org/10.6084/m9.figshare.33909865.
