# Supplementary material for: Distinct systemic metabolic phenotypes associated with blood culture positivity and bacterial etiology in community-acquired pneumonia (CAP)
Source: Front Cell Infect Microbiol. 2026 Sep 25;16:1924752. doi: 10.3389/fcimb.2026.1924752 (PMC13649496; doi:10.3389/fcimb.2026.1924752)
Supplement: Supplementary file 1 [file DataSheet1.docx]

**Distinct Systemic Metabolic Phenotypes Associated with Blood Culture Positivity and Bacterial Etiology in Community-Acquired Pneumonia (CAP)**

Mohammad M. Banoei^1,2^, Sachin Yende ^3, 4^, Derek C. Angus ^3, 4^, and Brent W. Winston^1,5^

1 Department of Critical Care Medicine, University of Calgary, Calgary, AB, Canada.

2 Department of Bio-Medical Engineering, University of Calgary, Calgary, AB, Canada.

3 Department of Critical Care Medicine, University of Pittsburgh, Pittsburgh, PA, USA.

4 Center for Research, Investigation, and Systems Modeling of Acute Illness, School of Medicine, University of Pittsburgh, Pittsburgh, PA, USA

5 Departments of Medicine and Biochemistry and Molecular Biology, University of Calgary, Calgary, AB, Canada

**Address correspondence to:**

Brent W. Winston, MD, Departments of Critical Care Medicine, Medicine and Biochemistry and Molecular Biology, University of Calgary, Health Research Innovation Center (HRIC), Room 4C64, 3280 Hospital Drive N.W., Calgary, Alberta, Canada, T2N 4Z6.

Tel: (403) 220-4331

Fax: (403) 283-1267

Email: [bwinston@ucalgary.ca](mailto:bwinston@ucalgary.ca)

Running Title: Plasma Metabolomics in bacterial CAP Pneumonia

**Figure S1.** Principal component analysis (PCA) score plots showing the overall structure and variability of metabolomics data across samples between culture positive and culture negative. Two-dimensional (top panels) and three-dimensional (bottom panels) PCA score plots are shown for each dataset, with samples coloured by culture status (green: positive; pink: negative). PCA was used as an unsupervised exploratory analysis to assess global variance patterns, sample distribution, and potential outliers rather than to model group separation. Shaded ellipses represent 95% confidence regions, and the percentage of variance explained by each principal component is indicated on the axes.

**Figure S2.** ^1^H-NMR based variable importance in projection (VIP) scores from the OPLS-DA model showing metabolites with VIP > 1.0, indicating significant contribution to group discrimination positive culture (positive) and negative culture (negative). Metabolites are ranked according to their VIP values, with higher scores reflecting greater influence on class separation. The adjacent heatmap displays relative metabolite abundance across groups (red = higher, blue = lower), illustrating the directionality of changes for the most discriminative features.

**Figure S3.** LC-DIMS/MS-based variable importance in projection (VIP) scores (coefficient plot) from the OPLS-DA model showing metabolites with VIP > 1.0, indicating significant contribution to group discrimination, culture-positive (positive) and culture-negative (negative). Metabolites are ranked according to their VIP values, with higher scores reflecting greater influence on class separation. The adjacent heatmap displays relative metabolite abundance across groups (red = higher, blue = lower), illustrating the directionality of changes for the most discriminative features.

**Figure S4.** GC-MS based variable importance in projection (VIP) scores (coefficient plot) from the OPLS-DA model showing metabolites with VIP > 1.0, indicating significant contribution to group discrimination positive culture (positive) and negative culture (negative). Metabolites are ranked according to their VIP values, with higher scores reflecting greater influence on class separation. The adjacent heatmap displays relative metabolite abundance across groups (red = higher, blue = lower), illustrating the directionality of changes for the most discriminative features.

| **Name** | **Mean (SD) of Culture Negative** | **Mean (SD) of Culture Positive** | **p-value** | **Positive culture/Negative Culture** |
| --- | --- | --- | --- | --- |
| Arginine | 0.344 (0.868) | -0.327 (1.017) | 0.0019 | Down |
| Formate | -0.332 (0.988) | 0.316 (0.915) | 0.0028 | Up |
| Valine | 0.266 (1.009) | -0.253 (0.935) | 0.018 | Down |
| Isoleucine | 0.242 (0.855) | -0.230 (1.081) | 0.0319 | Down |
| Citrate | -0.577 (1.079) | 0.549 (0.483) | < 0.0001 (W) | Up |
| 2-Aminobutyrate | 0.388 (0.787) | -0.370 (1.049) | 0.0006 (W) | Down |
| 2-Oxoglutarate | 0.243 (1.057) | -0.231 (0.894) | 0.0036 (W) | Down |
| Isopropanol | 0.255 (0.968) | -0.243 (0.980) | 0.0052 (W) | Down |
| Creatinine | -0.233 (1.047) | 0.222 (0.911) | 0.0076 (W) | Up |
| Phenylalanine | -0.283 (0.958) | 0.269 (0.975) | 0.0153 (W) | Up |
| Lysine | 0.136 (1.172) | -0.129 (0.797) | 0.0264 (W) | Down |
| Hypoxanthine | -0.216 (1.048) | 0.206 (0.917) | 0.0317 (W) | Up |
| Isobutyrate | -0.132 (1.093) | 0.126 (0.898) | 0.0320 (W) | Up |
| Ornithine | 0.217 (1.022) | -0.206 (0.945) | 0.0422 (W) | Down |

**Table S1.** ^1^H-NMR-based differential metabolites between culture-positive and culture-negative groups. The table presents mean (SD) normalized metabolite levels in each group, corresponding p-values, and direction of change in the culture-positive group relative to culture-negative (Up = higher in culture-positive; Down = lower in culture-positive). P-values marked with (W) indicate Wilcoxon rank-sum test. Only metabolites meeting statistical significance (p < 0.05) are shown.

**Figure S4.** ^1^H-NMR-based box-and-whisker plots showing the distribution of significantly altered metabolites between culture-positive and culture-negative groups. Each panel represents one metabolite. Boxes indicate the interquartile range (IQR) with the median shown as the central line; whiskers represent the data range, and individual points correspond to individual samples. Differences between groups reflect the direction and magnitude of metabolite changes contributing to group discrimination.

| **Name** | **Mean (SD) of Culture Negative** | **Mean (SD) of Culture Positive** | **p-value** | **Culture Negative/Culture Positive** |
| --- | --- | --- | --- | --- |
| PC ae C40:5 | 0.335 (0.949) | -0.343 (0.942) | 0.0018 | Up |
| lysoPC a C20:4 | 0.313 (0.785) | -0.321 (1.100) | 0.0039 | Up |
| C14:1 | 0.295 (0.883) | -0.302 (1.033) | 0.0065 | Up |
| C8:1 | -0.292 (0.963) | 0.299 (0.959) | 0.007 | Down |
| C3 | -0.288 (0.939) | 0.295 (0.986) | 0.008 | Down |
| C0 | -0.274 (0.954) | 0.281 (0.978) | 0.0115 | Down |
| C10:2 | -0.274 (0.839) | 0.281 (1.082) | 0.0117 | Down |
| PC aa C32:1 | -0.267 (0.958) | 0.274 (0.978) | 0.014 | Down |
| PC aa C32:2 | -0.256 (1.020) | 0.262 (0.919) | 0.0187 | Down |
| C6 (C4:1-DC) | -0.235 (0.965) | 0.241 (0.989) | 0.0316 | Down |
| SM C18:0 | 0.233 (0.994) | -0.239 (0.960) | 0.0327 | Up |
| PC ae C40:1 | 0.232 (0.820) | -0.237 (1.117) | 0.0339 | Up |
| PC aa C34:3 | -0.223 (0.826) | 0.229 (1.116) | 0.0413 | Down |
| PC aa C36:1 | -0.222 (0.966) | 0.227 (0.995) | 0.0425 | Down |
| PC aa C32:0 | -0.347 (0.834) | 0.356 (1.039) | 0.0002 (W) | Down |
| PC aa C34:1 | -0.300 (0.971) | 0.307 (0.945) | 0.0014 (W) | Down |
| PC aa C30:0 | -0.276 (0.999) | 0.283 (0.930) | 0.0047 (W) | Down |
| PC ae C38:4 | 0.299 (0.765) | -0.307 (1.122) | 0.0047 (W) | Up |
| lysoPC a C18:2 | 0.289 (0.920) | -0.296 (1.003) | 0.0051 (W) | Up |
| PC ae C40:4 | 0.270 (0.965) | -0.276 (0.970) | 0.0063 (W) | Up |
| PC aa C42:5 | 0.242 (0.867) | -0.248 (1.076) | 0.0098 (W) | Up |
| C3-DC (C4-OH) | -0.211 (1.083) | 0.216 (0.868) | 0.0126 (W) | Down |
| Phenylalanine | -0.276 (0.704) | 0.283 (1.175) | 0.0126 (W) | Down |
| SM (OH) C16:1 | 0.262 (0.947) | -0.268 (0.993) | 0.0209 (W) | Up |
| PC ae C36:0 | -0.277 (0.833) | 0.284 (1.084) | 0.0282 (W) | Down |
| C5 | -0.231 (1.016) | 0.237 (0.937) | 0.0433 (W) | Down |
| PC ae C38:3 | 0.206 (0.999) | -0.211 (0.969) | 0.0443 (W) | Up |
| lysoPC a C17:0 | 0.184 (0.968) | -0.189 (1.009) | 0.0485 (W) | Up |

**Table S2.** LC-DIMS/MS-based differential metabolites between culture-positive and culture-negative groups. The table presents mean (SD) normalized metabolite levels in each group, corresponding p-values, and direction of change in the culture-positive group relative to culture-negative (Up = higher in culture-positive; Down = lower in culture-positive). P-values marked with (W) indicate Wilcoxon rank-sum test. Only metabolites meeting statistical significance (p < 0.05) are shown.

**Figure S5.** LC-DIMS/MS-based box-and-whisker plots showing the distribution of significantly altered metabolites between culture-positive and culture-negative groups. Each panel represents one metabolite. Boxes indicate the interquartile range (IQR) with the median shown as the central line; whiskers represent the data range, and individual points correspond to individual samples. Differences between groups reflect the direction and magnitude of metabolite changes contributing to group discrimination.

| **Name** | **Mean (SD) of** Culture **Negative** | **Mean (SD) of** Culture **Positive** | **p-value** | Culture **Negative/**Culture **Positive** |
| --- | --- | --- | --- | --- |
| Unkwnown | 0.348 (0.905) | -0.340 (0.981) | 0.0016 | Down |
| Unkwnown | 0.320 (0.897) | -0.313 (1.006) | 0.0038 | Down |
| Unkwnown | -0.266 (0.952) | 0.260 (0.988) | 0.0169 | up |
| Glyceric acid (3TMS) | 0.259 (0.900) | -0.253 (1.038) | 0.0202 | Down |
| Unkwnown | -0.226 (1.189) | 0.221 (0.722) | 0.0453 | Up |
| Heptadecanoic acid (1TMS) | 0.434 (0.880) | -0.424 (0.934) | < 0.0001 | Down |
| Unkwnown | -0.371 (1.033) | 0.362 (0.829) | 0.0001 (W) | Up |
| Tetradecanoic acid (1TMS) | 0.161 (0.959) | -0.157 (1.026) | 0.0001 (W) | Down |
| Unkwnown | 0.466 (0.831) | -0.454 (0.948) | 0.0002 (W) | Down |
| Unkwnown | 0.386 (0.901) | -0.376 (0.956) | 0.0003 (W) | Down |
| Octadecadienoic acid, 9,12-(Z,Z)- (1TMS) | 0.309 (1.020) | -0.302 (0.892) | 0.0003 (W) | Down |
| Pyruvic acid (1MEOX) (1TMS)) | 0.353 (1.014) | -0.344 (0.866) | 0.0004 (W) | Down |
| Pentadecane, n- | 0.286 (0.889) | -0.279 (1.033) | 0.0007 (W) | Down |
| Unkwnown | -0.372 (0.902) | 0.363 (0.966) | 0.0011 (W) | Up |
| Unkwnown | -0.251 (1.066) | 0.245 (0.876) | 0.0014 (W) | Up |
| Unkwnown | 0.266 (0.868) | -0.259 (1.061) | 0.0023 (W) | Down |
| Alanine (2TMS) | 0.317 (0.716) | -0.310 (1.141) | 0.0034 (W) | Down |
| Unkwnown | 0.272 (0.632) | -0.265 (1.210) | 0.0038 (W) | Down |
| Unkwnown | 0.223 (0.581) | -0.217 (1.254) | 0.0062 (W) | Down |
| Unkwnown | -0.272 (0.836) | 0.266 (1.083) | 0.0065 (W) | Up |
| Unkwnown | -0.296 (0.822) | 0.288 (1.081) | 0.0067 (W) | Up |
| Urea (2TMS) | -0.284 (0.965) | 0.277 (0.965) | 0.0076 (W) | Up |
| Unkwnown | 0.384 (1.010) | -0.375 (0.845) | 0.0090 (W) | Down |
| Heptadecane, n- | -0.359 (0.903) | 0.350 (0.975) | 0.0090 (W) | Up |
| Unkwnown | 0.422 (0.991) | -0.411 (0.832) | 0.0095 (W) | Down |
| Unkwnown | 0.102 (1.270) | -0.100 (0.638) | 0.0098 (W) | Down |
| Unkwnown | 0.237 (1.064) | -0.232 (0.886) | 0.0107 (W) | Down |
| Unkwnown | 0.361 (1.016) | -0.352 (0.858) | 0.0109 (W) | Down |
| Unkwnown | 0.260 (0.661) | -0.254 (1.200) | 0.0111 (W) | Down |
| Unkwnown | 0.289 (0.633) | -0.282 (1.202) | 0.0116 (W) | Down |
| Unkwnown | 0.365 (1.014) | -0.356 (0.858) | 0.0125 (W) | Down |
| Unkwnown | 0.367 (0.997) | -0.358 (0.874) | 0.0154 (W) | Down |
| Ornithine (3TMS) (Derivate not found) | 0.177 (0.918) | -0.173 (1.056) | 0.0169 (W) | Down |
| Octadecenoic acid, 9-(E)- (1TMS) | -0.260 (1.083) | 0.253 (0.851) | 0.0179 (W) | Up |
| Uric acid (4TMS) | -0.133 (0.818) | 0.130 (1.145) | 0.0183 (W) | Up |
| Unkwnown | -0.210 (1.101) | 0.205 (0.855) | 0.0188 (W) | UP |
| Unkwnown | 0.334 (0.835) | -0.326 (1.049) | 0.0194 (W) | Down |
| Unkwnown | 0.237 (1.064) | -0.232 (0.886) | 0.0198 (W) | Down |
| Unkwnown | 0.264 (0.898) | -0.258 (1.037) | 0.0231 (W) | Down |
| Unkwnown | 0.162 (1.174) | -0.158 (0.778) | 0.0237 (W) | Down |
| Arabinonic acid (5TMS) | 0.257 (0.651) | -0.250 (1.207) | 0.0256 (W) | Down |
| Sulfuric acid (2TMS)) | -0.156 (1.023) | 0.152 (0.965) | 0.0261 (W) | Up |
| Unkwnown | -0.244 (0.966) | 0.238 (0.987) | 0.0269 (W) | Up |
| Unkwnown | 0.291 (0.939) | -0.284 (0.986) | 0.0281 (W) | Down |
| Unkwnown | 0.300 (1.105) | -0.292 (0.795) | 0.0342 (W) | Down |
| Urea (2TMS)) | -0.241 (0.839) | 0.235 (1.095) | 0.0423 (W) | Up |
| Inositol, myo- (6TMS) | -0.235 (0.840) | 0.229 (1.097) | 0.0453 (W) | Up |
| Unkwnown | 0.240 (0.919) | -0.234 (1.031) | 0.0464 (W) | Down |
| Phosphoric acid (3TMS)) | -0.201 (1.007) | 0.196 (0.966) | 0.0464 (W) | Up |
| Alanine (2TMS)) | 0.361 (1.035) | -0.352 (0.835) | 0.0468 (W) | Down |
| Valine (2TMS) | 0.056 (1.131) | -0.055 (0.864) | 0.0474 (W) | Down |

**Table S3.** GC-MS-based differential metabolites between culture-positive and culture-negative groups. The table presents mean (SD) normalized metabolite levels in each group, corresponding p-values, and direction of change in the culture-positive group relative to culture-negative (Up = higher in culture-positive; Down = lower in culture-positive). P-values marked with (W) indicate Wilcoxon rank-sum test. Only metabolites meeting statistical significance (p < 0.05) are shown.

**Figure S6.** GC-MS-based box-and-whisker plots showing the distribution of significantly altered metabolites between culture-positive and culture-negative groups. Each panel represents one metabolite. Boxes indicate the interquartile range (IQR) with the median shown as the central line; whiskers represent the data range, and individual points correspond to individual samples. Differences between groups reflect the direction and magnitude of metabolite changes contributing to group discrimination

**Figure S7.** Principal component analysis (PCA) score plots showing the overall structure and variability of metabolomics data across samples culture positive and culture negative. Two-dimensional (top panels) and three-dimensional (bottom panels) PCA score plots are shown for each dataset, with samples colored by culture status (green: positive; pink: negative). PCA was used as an unsupervised exploratory analysis to assess global variance patterns, sample distribution, and potential outliers rather than to model group separation. Shaded ellipses represent 95% confidence regions, and the percentage of variance explained by each principal component is indicated on the axes.

**Figure S8.** ^1^H-NMR-based variable importance in projection (VIP) scores (coefficient plot) from the OPLS-DA model showing metabolites with VIP > 1.0, indicating significant contribution to group discrimination between S. pneumonia and other bacteria. Metabolites are ranked according to their VIP values, with higher scores reflecting greater influence on class separation. The adjacent heatmap displays relative metabolite abundance across groups (red = higher, blue = lower), illustrating the directionality of changes for the most discriminative features.

**Figure S9.** LC-DIMS/MS-based variable importance in projection (VIP) scores (coefficient plot) from the OPLS-DA model showing metabolites with VIP > 1.0, indicating significant contribution to group discrimination between *S. pneumoniae* and other bacterial causes. Metabolites are ranked according to their VIP values, with higher scores reflecting greater influence on class separation. The adjacent heatmap displays relative metabolite abundance across groups (red = higher, blue = lower), illustrating the directionality of changes for the most discriminative features.

**Figure S10.** GC-MS-based variable importance in projection (VIP) scores from the OPLS-DA model showing metabolites with VIP > 1.0, indicating significant contribution to group discrimination *S. pneumonia* and other bacterial causes. Metabolites are ranked according to their VIP values, with higher scores reflecting greater influence on class separation. The adjacent heatmap displays relative metabolite abundance across groups (red = higher, blue = lower), illustrating the directionality of changes for the most discriminative features.

| **Name** | **Mean (SD) of Other bacterial causes** | **Mean (SD) of *S. pneumoniae*** | **p-value** | **Other bacterial causes/*s. pneumonia*** |
| --- | --- | --- | --- | --- |
| 2-Hydroxybutyrate | -0.268 (0.916) | 0.536 (0.974) | 0.0122 | Up |
| 2-Aminobutyrate | -0.246 (1.060) | 0.493 (0.653) | 0.022 | Up |
| 2-Oxoisocaproate | -0.219 (1.030) | 0.437 (0.801) | 0.0437 | Up |
| Isobutyrate | -0.281 (0.915) | 0.561 (0.953) | 0.0041 (W) | Up |
| Phenylalanine | -0.138 (1.089) | 0.276 (0.755) | 0.0317 (W) | Up |

**Table S4.** ¹H-NMR-based differential metabolites between *S. pneumoniae* and other bacterial causes. The table presents mean (SD) normalized metabolite levels in each group, corresponding p-values, and direction of change in the *S. pneumoniae* group relative to other bacterial causes (Up = higher in *S. pneumoniae*; Down = lower in *S. pneumoniae*). P-values marked with (W) indicate Wilcoxon rank-sum test. Only metabolites meeting statistical significance (p < 0.05) are shown.

**Figure S11.** ^1^H-NMR-based box-and-whisker plots showing the distribution of significantly altered metabolites between s. pneumonia and other bacteria. Each panel represents one metabolite. Boxes indicate the interquartile range (IQR) with the median shown as the central line; whiskers represent the data range, and individual points correspond to individual samples. Differences between groups reflect the direction and magnitude of metabolite changes contributing to group discrimination.

| **Name** | **Mean (SD) of Other bacterial causes** | **Mean (SD) of *S. pneumoniae*** | **p-value** | **Other bacterial causes/*S. pneumoniae*** |
| --- | --- | --- | --- | --- |
| C18 | 0.274 (0.990) | -0.509 (0.828) | 0.0161 | Down |
| PC ae C34:0 | -0.266 (0.966) | 0.494 (0.895) | 0.0199 | Up |
| PC aa C34:2 | -0.257 (0.864) | 0.478 (1.088) | 0.0244 | Up |
| PC aa C36:2 | -0.247 (1.005) | 0.458 (0.843) | 0.0317 | Up |
| PC aa C34:1 | -0.246 (0.978) | 0.457 (0.902) | 0.032 | Up |
| PC aa C32:1 | -0.236 (0.883) | 0.439 (1.087) | 0.04 | Up |
| PC ae C40:5 | 0.229 (0.981) | -0.425 (0.922) | 0.0472 | Down |
| PC ae C42:4 | 0.187 (1.116) | -0.348 (0.635) | 0.0108 (W) | Down |
| PC ae C42:3 | 0.212 (1.064) | -0.393 (0.752) | 0.0288 (W) | Down |
| C3-DC (C4-OH) | -0.232 (0.938) | 0.432 (1.000) | 0.0334 (W) | Up |
| PC ae C42:2 | 0.195 (1.074) | -0.363 (0.750) | 0.0360 (W) | Down |
| Serine | 0.215 (1.109) | -0.399 (0.607) | 0.0478 (W) | Down |

**Table S5.** LC-DIMS/MS-based differential metabolites between s. pneumonia and other bacteria. The table presents mean (SD) normalized metabolite levels in each group, corresponding p-values, and direction of change in the culture-positive group relative to culture-negative (Up = higher in culture-positive; Down = lower in culture-positive). P-values marked with (W) indicate Wilcoxon rank-sum test. Only metabolites meeting statistical significance (p < 0.05) are shown.

**Figure S12.** LC-DIMS/MS-based box-and-whisker plots showing the distribution of significantly altered metabolites between *S. pneumoniae* and other bacterial causes. Each panel represents one metabolite. Boxes indicate the interquartile range (IQR) with the median shown as the central line; whiskers represent the data range, and individual points correspond to individual samples. Differences between groups reflect the direction and magnitude of metabolite changes contributing to group discrimination.

| **Name** | **Mean (SD) of Other bacterial causes** | **Mean (SD) of *S. pneumoniae*** | **p-value** | **Other Bacterial causes/ *S. pneumoniae*** |
| --- | --- | --- | --- | --- |
| Unknown | 0.298 (0.795) | -0.641 (1.121) | 0.0038 | Down |
| Octadecenoic acid, 9-(E)- (1TMS) | -0.232 (1.041) | 0.500 (0.705) | 0.0272 | Up |
| Butanoic acid, 3-hydroxy- (2TMS) | -0.221 (0.974) | 0.475 (0.917) | 0.0363 | Up |
| Inositol, myo- (6TMS) | -0.216 (0.917) | 0.465 (1.048) | 0.0408 | Up |
| Butanoic acid, 2-hydroxy- (2TMS) | -0.305 (1.036) | 0.657 (0.486) | 0.0002 (W) | Up |
| Inositol-2-phosphate, myo- (7TMS) | -0.317 (0.878) | 0.683 (0.926) | 0.0039 (W) | Up |
| Unknown | 0.282 (0.764) | -0.608 (1.197) | 0.0043 (W) | Down |
| Tetradecanoic acid (1TMS) | 0.223 (0.626) | -0.479 (1.445) | 0.0083 (W) | Down |
| Unknown | -0.275 (1.023) | 0.592 (0.652) | 0.0099 (W) | Up |
| Unknown | 0.257 (0.877) | -0.554 (1.055) | 0.0118 (W) | Down |
| Unknown | 0.241 (0.825) | -0.518 (1.173) | 0.0118 (W) | Down |
| Unknown | -0.240 (1.017) | 0.517 (0.762) | 0.0140 (W) | Up |
| Uric acid (4TMS) | -0.165 (1.124) | 0.355 (0.541) | 0.0210 (W) | Up |
| Glutamic acid (3TMS) | 0.174 (1.019) | -0.376 (0.881) | 0.0227 (W) | Down |
| Unknown | -0.206 (1.084) | 0.443 (0.615) | 0.0245 (W) | Up |
| Unknown | -0.197 (1.053) | 0.425 (0.743) | 0.0286 (W) | Up |
| Aspartic acid (3TMS) | -0.220 (1.030) | 0.475 (0.769) | 0.0286 (W) | Up |
| Unknown | -0.262 (0.976) | 0.565 (0.826) | 0.0331 (W) | Up |
| Unknown | 0.092 (1.079) | -0.199 (0.807) | 0.0331 (W) | Down |
| Phosphoric acid monomethyl ester (2TMS) | 0.213 (0.965) | -0.458 (0.951) | 0.0383 (W) | Down |
| Benzoic acid, (1TMS) | 0.222 (1.018) | -0.479 (0.799) | 0.0383 (W) | Down |
| Unknown | -0.257 (1.073) | 0.553 (0.509) | 0.0473 (W) | Up |

**Table S6.** GC-MS-based differential metabolites between s. pneumonia and other bacteria. The table presents mean (SD) normalized metabolite levels in each group, corresponding p-values, and direction of change in the culture-positive group relative to culture-negative (Up = higher in culture-positive; Down = lower in culture-positive). P-values marked with (W) indicate Wilcoxon rank-sum test. Only metabolites meeting statistical significance (p < 0.05) are shown.

**Figure S13.** GC-MS-based box-and-whisker plots showing the distribution of significantly altered metabolites between *S. pneumoniae* and other bacterial causes. Each panel represents one metabolite. Boxes indicate the interquartile range (IQR) with the median shown as the central line; whiskers represent the data range, and individual points correspond to individual samples. Differences between groups reflect the direction and magnitude of metabolite changes contributing to group discrimination.

**Figure S14.** ^1^H-NMR-based pairwise correlation matrix of detected metabolites across all samples. The heatmap displays correlation coefficients (Spearman/Pearson, as specified in Methods) between metabolites, with red indicating positive correlations and blue indicating negative correlations; color intensity reflects the strength of association. The diagonal represents self-correlation (r = 1). Hierarchical clustering groups metabolites with similar correlation patterns, revealing coordinated metabolic modules and potential pathway-level interactions.

**Figure S15.** LC-DIMS/MS-based pairwise correlation matrix of detected metabolites across all samples. The heatmap displays correlation coefficients (Spearman/Pearson, as specified in Methods) between metabolites, with red indicating positive correlations and blue indicating negative correlations; color intensity reflects the strength of association. The diagonal represents self-correlation (r = 1). Hierarchical clustering groups metabolites with similar correlation patterns, revealing coordinated metabolic modules and potential pathway-level interactions.

**Figure S16.** GC-MS-based pairwise correlation matrix of detected metabolites across all samples. The heatmap displays correlation coefficients (Spearman/Pearson, as specified in Methods) between metabolites, with red indicating positive correlations and blue indicating negative correlations; color intensity reflects the strength of association. The diagonal represents self-correlation (r = 1). Hierarchical clustering groups metabolites with similar correlation patterns, revealing coordinated metabolic modules and potential pathway-level interactions.

| **Platform** | **Culture-positive median \|ρ\|** | **Culture-negative median \|ρ\|** | **Main interpretation** |
| --- | --- | --- | --- |
| ¹H-NMR | **0.124** | 0.087 | modestly stronger |
| LC-DI-MS/MS | **0.350** | 0.255 | clearly stronger |
| GC-MS | **0.202** | 0.193 | slightly stronger |

**Table S7. Comparison of metabolite–clinical correlation strength between culture-positive and culture-negative CAP patients.** Spearman correlations were calculated separately within culture-positive and culture-negative groups for metabolites/features included in the main metabolite–clinical correlation analysis. Median absolute correlation coefficients (|ρ|) and the proportions of moderate (|ρ| ≥ 0.30) and strong (|ρ| ≥ 0.40) correlations are summarized by analytical platform and clinical variable. These stratified analyses were exploratory and were not intended as formal tests of interaction between culture status and clinical severity.
